# Update in the Early Management and Reperfusion Strategies of Patients with Acute Ischemic Stroke

**DOI:** 10.1155/2018/9168731

**Published:** 2018-06-28

**Authors:** Aldo A. Mendez, Edgar A. Samaniego, Sunil A. Sheth, Sudeepta Dandapat, David M. Hasan, Kaustubh S. Limaye, Bradley J. Hindman, Colin P. Derdeyn, Santiago Ortega-Gutierrez

**Affiliations:** ^1^Department of Neurology, University of Iowa Hospitals and Clinics, Iowa City, IA, USA; ^2^Department of Neurosurgery, University of Iowa Hospitals and Clinics, Iowa City, IA, USA; ^3^Department of Radiology, University of Iowa Hospitals and Clinics, Iowa City, IA, USA; ^4^Department of Neurology and Neurosurgery, The University of Texas Health Science Center at Houston, Houston, TX, USA; ^5^Department of Anesthesia, University of Iowa Hospitals and Clinics, Iowa City, IA, USA

## Abstract

Acute ischemic stroke (AIS) remains a leading cause of death and long-term disability. The paradigms on prehospital care, reperfusion therapies, and postreperfusion management of patients with AIS continue to evolve. After the publication of pivotal clinical trials, endovascular thrombectomy has become part of the standard of care in selected cases of AIS since 2015. New stroke guidelines have been recently published, and the time window for mechanical thrombectomy has now been extended up to 24 hours. This review aims to provide a focused up-to-date review for the early management of adult patients with AIS and introduce the new upcoming areas of ongoing research.

## 1. Introduction

Stroke ranks number five among all causes of death in the United States (US) and is also a leading cause of serious long-term disability. On average, every 40 seconds, someone in the United States has a stroke and, every 4 minutes, someone dies of stroke. Stroke costs at least $70 billion each year in the US. World-wide, stroke is the second leading cause of death. Of all strokes, 87% are ischemic [1]. Given the massive social and economic burden that ischemic stroke represents, prevention and acute management of this disease is of paramount importance.

In acute stroke, ischemia is rarely complete at presentation. Residual perfusion, which depends on collateral vessels and local perfusion pressures, creates a region, called the penumbra, in which residual perfusion attempts to supply sufficient oxygen to maintain a close to normal tissue concentration of ATP with some degree of energy failure [2]. In contrast to areas of benign oligemia, the penumbra is an ischemic, but malfunctioning, living brain tissue that will die unless the blood supply is restored [3]. Acute stroke management, including reperfusion therapies, is aimed at restoring adequate blood supply to these areas at risk of infarction.

**Figure 1:**
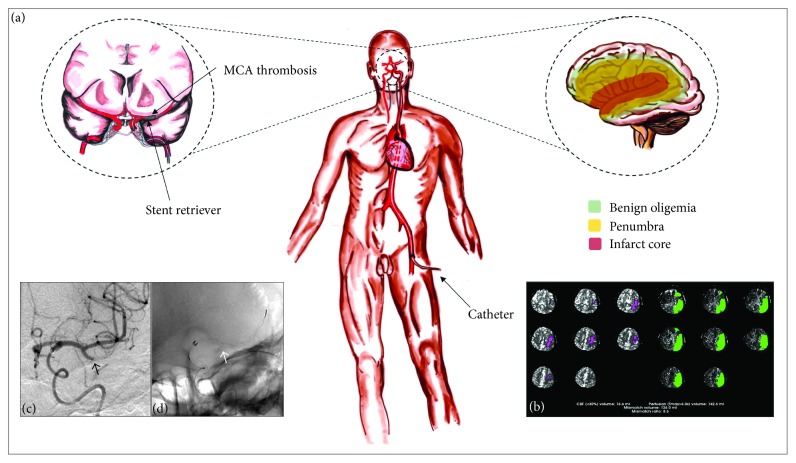
(a) Schematic representation of one of the most common endovascular techniques using a stent retriever to treat an acute left middle cerebral artery stroke secondary to an LVO presenting at 12 hours. (b) Identification of infarct core and potentially salvageable tissue using automated software (RAPID). (c, d) Angiogram demonstrating L MCA occlusion (black arrow) and stent retriever deployment (white arrow).

Until recently, intravenous alteplase administered within 3–4.5 hours after symptom onset was the only reperfusion therapy with proven efficacy in patients with acute ischemic stroke. However, after the publication of five pivotal clinical trials [4–8], endovascular thrombectomy is accepted as the standard of care for patients with large vessel occlusion (LVO) in the anterior circulation [9]. Although the initial trials indicated that endovascular thrombectomy did not confer benefit when reperfusion was not accomplished within 6-7 hours, two recent trials, DAWN [10] and DEFUSE 3 [11], have demonstrated that the window for endovascular thrombectomy can, in some patients, be extended up to 16–24 hours from last known normal using perfusion imaging. New stroke guidelines have been published to incorporate these findings and the potential time window for mechanical thrombectomy has now been extended up to 24 hours [9].  Figure 1 depicts one of the most common endovascular techniques using a stent retriever to treat acute ischemic stroke secondary to an LVO presenting at 12 hours. The aim of this manuscript is to provide a focused up-to-date review for the early management of adult patients with acute arterial ischemic stroke and introduce the new upcoming areas of ongoing research.

## 2. Prehospital Care

The use of Emergency Medical Services has been associated with earlier hospital arrival and more rapid treatment [12]. The primary goals of EMS in acute stroke are rapid evaluation, triage, and transport to a stroke-ready hospital. Current guidelines prioritize supplemental oxygen to maintain adequate oxygen saturations (SpO_2_ > 94%), determination of glucose level, and treatment if <60 mg/dL to rule out a potential stroke mimic. EMS may also establish large bore IV access and obtain blood samples for laboratory testing en route. Although these recommendations represent an ideal scenario, it is critical that these interventions do not delay transport of the patient to the hospital [9]. The most important reason for missing recanalization therapy is time delay in the prehospital phase [13].

Obtaining information prior to hospital arrival can assist in the prehospital diagnosis of stroke or stroke mimic using stroke assessment systems, assess comorbidities, medications, and recent trauma or surgeries that could contraindicate the use of IV tPA. However, the most important piece of information necessary for potential reperfusion therapy is the time the patient was last known normal. The patient should then be promptly triaged and transported to the nearest facility with reperfusion therapy capabilities [9]. Also, prehospital providers should notify the hospital about pending stroke patient arrival, as this has been associated with significant reduction in stroke time targets and tPA administration [9, 14].

**Figure 2:**
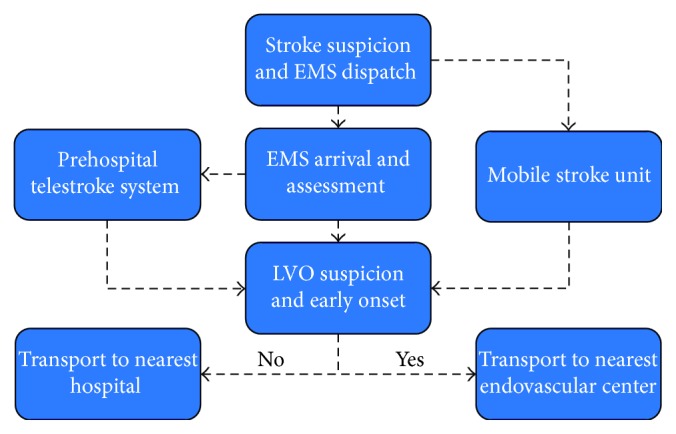
Prehospital stroke algorithm paradigm.

Current guidelines recommend patient transportation to the nearest hospital with tPA capacity [9]. This, however, may be detrimental for patients with LVO because of the time delay associated with established “drip and ship” models [15]. IV tPA results in a low recanalization rate of patients with LVO occlusion [16]. A study by Mokin et al. [17] demonstrated that one out of three patients with LVO with initial favorable imaging profile became ineligible for endovascular thrombectomy during interhospital transfer based on ASPECTS criteria. In this study, except for NIHSS severity, no other baseline factors could identify which patients were at risk for ASPECTS deterioration during interhospital transfer. In the SWIFT PRIME trial, when comparing the outcomes in patients treated under the current drip and ship paradigm versus primary endovascular center presentation, outcomes were significantly worse for those patients who were transferred to the center with endovascular thrombectomy capabilities after receiving IV tPA at the outside hospital [6]. In the current era of endovascular therapy, current prehospital stroke evaluation should include stroke severity and not only stroke recognition. Triaging severe cases directly to endovascular therapy-capable center may provide the best opportunity to optimize endovascular thrombectomy [18]. To address this matter, several approaches for the early recognition of LVO have been developed. These include prehospital stroke scales to be used by prehospital personnel in the field such as the 3ISS (3-Item Stroke Scale) [19], LAMS (Los Angeles Motor Scale) [20], RACE (Rapid Arterial Occlusion Evaluation Scale) [21], CPSSS (Cincinnati Prehospital Stroke Severity Scale) [22], and PASS (Prehospital Acute Stroke Severity) [23], as well as Mobile Stroke Units (MSU), and telemedicine. Current guidelines integrate these findings and recommend (Class IIb) that when several facilities with tPA capabilities exist within a specific region, the benefit of bypassing the nearest facility to transfer the patient to one that offers a higher level of stroke care located within a reasonable distance, including mechanical thrombectomy, may be considered [9]. RACECAT (Direct Transfer to an Endovascular Center Compared to Transfer to the Closest Stroke Center in Acute Stroke Patients with Suspected Large Vessel Occlusion) is an ongoing prospective, multicenter, cluster randomized controlled trial occurring in Spain. In this study, two strategies in acute stroke patients with suspected acute LVO identified by EMS at first assessment in the field will be compared: transfer to the closest local stroke center versus direct transfer to an endovascular stroke center. In order to maximize the sensitivity and specificity of LVO diagnosis, EMS will utilize the RACE scale (Rapid Arterial oCclusion Evaluation) as a prehospital screening tool to identify acute stroke patients with suspicion and will contact a stroke neurologist on call using a prehospital telestroke system within the ambulance, who will confirm inclusion criteria for LVO and will allocate the subjects to a specific intervention according to a preestablished temporal sequence.  Figure 2 depicts a potential alternative to current prehospital stroke paradigms that will need to be elucidated in the near future.

An alternative approach to improve the triage and treatment process has occurred through the implementation of Mobile Stroke Units (MSU) with imaging capabilities in large urban areas. The BEST-MSU (Benefits of Stroke Treatment Delivered Using a Mobile Stroke Unit) trial was launched to compare stroke management using a MSU versus standard management. So far, the run-in phase of this study has provided essential information to help in the final design of their study. They have also shown that their average door-to-needle time (25 minutes) on the MSU is comparable with the fastest ED door-to-needle times reported in the literature [24]. Another study by the Cleveland Clinic compared the evaluation and treatment of patients on a Mobile Stroke Unit, using telemedicine for physician presence, against a control group of patients brought to the emergency department through ambulance. The time from door to CT completion (13 minutes (IQR, 9–21 minutes) versus 18 minutes (IQR, 12–26 minutes)) and from door to IV tPA (32 minutes (IQR, 24–47 minutes) versus 58 minutes (IQR, 53–68 minutes)) was significantly shorter in the MSU compared with the control group. This study showed the feasibility in performing prehospital stroke assessment and IV tPA therapy using a MSU with telemedicine capabilities [25]. Some studies have suggested that MSU systems can be cost-effective, especially when reducing the number of staff within the unit by using telemedicine [26, 27]. The efficiency of these systems, however, is related to population density, which may limit its benefits in rural areas [26].

Parallel with the development of reperfusion therapies, several measures are underway to optimize the prehospital stroke rescue chain. Measures for improvement include continuous public awareness campaigns; education of emergency medical service personnel; the use of standardized, validated scales for recognition of stroke symptoms and for triaging to the appropriate institution; advance notification to the receiving hospital; mobile CT-equipped ambulances directed by an onboard stroke neurologist or telemedicine consultation; and blood biomarkers [28–30]. Prompt assessment and adequate triaging of patients with acute ischemic stroke is crucial for timely delivery of reperfusion therapies and optimize outcome.

## 3. Intravenous Thrombolytics

In 1995, the National Institute of Neurological Disorders and Stroke (NINDS) trials showed the benefit of using intravenous (IV) tissue plasminogen activator (tPA) over placebo within 3 hours of symptom onset [31]. Based on these results, in 1996, the Food and Drug Administration (FDA) approved the use of IV tPA (or alteplase) for patients with AIS presenting within 3 hours of symptom onset.  Table 1 lists the inclusion and exclusion criteria for the use of IV tPA within 3 h of symptom onset. In 2008, ECASS (European Cooperative Acute Stroke Study) III showed benefit of IV tPA over placebo among those treated within 3 to 4.5 hours of symptom onset [32, 33]. Although the FDA has not modified the use of IV tPA beyond the 3 hours window, the recent stroke guidelines from the American Heart Association (AHA) recommend using IV tPA up to 4.5 h from onset of symptoms in eligible patients: patients ≤ 80 years of age, without a history of both diabetes mellitus and stroke, with NIHSS score ≤ 25, not taking oral anticoagulation, and without radiologic evidence of ischemic injury involving more than one-third of the MCA territory [9, 34]. Delay in treatment reduces the opportunity of receiving reperfusion therapies and worsens neurological outcomes [35, 36]. A meta-analysis that included 3670 patients, described the therapeutic benefit and clinical risk of IV tPA in relation to time. In this analysis, the odds of a favorable 3-month outcome increased as onset to start of treatment decreased (*P*=0.0269). Adjusted odds of a favorable 3-month outcome were 2.55 (95% CI 1.44–4.52) for 0–90 min, 1.64 (1.12–2.40) for 91–180 min, 1.34 (1.06–1.68) for 181–270 min, and 1.22 (0.92–1.61) for 271–360 min in favor of the alteplase group. Based on these results, five patients need to be treated 0–90 min, nine patients 91–180 min, or 15 patients 181–270 min after symptom onset for one of them to have an excellent outcome (mRS score 0–1) attributable to treatment. No benefit of alteplase treatment was seen after around 270 min, and beyond 4.5 h the risk of using IV tPA might outweigh the benefit [36]. Of note, most of the patients included in this meta-analysis did not have an LVO. Other clinical trials have explored using low-dose tPA (0.6 mg/kg) as compared to the standard dose (0.9 mg/kg). Although they demonstrated less risk of intracerebral hemorrhage with low-dose tPA, they did not show noninferiority of low-dose tPA to the standard dose with respect to death and disability at 90 days [37]. More recently, the WAKE-UP (Efficacy and Safety of MRI-Based Thrombolysis in Wake-up Stroke) trial has shown that the administration of intravenous alteplase thrombolysis decreases functional disability at 3 months in patients with mild to moderate severity strokes of unknown time of onset, when patients were selected on the basis of simple MRI criteria showing a lesion on diffusion-weighted imaging but without a corresponding hyperintensity on fluid-attenuated inversion recovery (FLAIR) [38].

Despite recommendations to reduce the door-to-needle time to <60 minutes, fewer than one-third of patients treated with IV tPA received tPA within 60 minutes, and less than 5% of all stroke patients receive tPA at all [35, 39]. In addition to the narrow time window, IV tPA has numerous limitations. IV tPA has a low potential to recanalize occluded vessels with a large (>8 mm) thrombus [40], resulting in a poor recanalization rate (13% to 50%) in large vessel occlusion stroke and a low rate of benefit in the patients having the most disabling strokes [16]. To overcome these limitations, alternative therapies have been studied. Some of these alternatives that have been tested in clinical trials include (1) the use of systemic tenecteplase [41, 42], or desmolteplase [43, 44] or (2) the augmentation of systemic IV tPA recanalization with ultrasound. *Tenecteplase (TNK)* is a genetically engineered variant of tPA that has a longer half-life and is more fibrin specific than tPA. TNK has properties which make it a faster and more complete thrombolytic agent and, at the same time, with less bleeding complications and early reocclusions [45]. Furthermore, TNK can be given as a one-time bolus without need for an infusion [46]. In the Tenecteplase versus Alteplase for Acute Ischemic Stroke (TAAIS) trial, 75 patients, who arrived <6 h after the onset of ischemic stroke, were randomly assigned to receive either tPA (0.9 mg/kg) or TNK (0.1 mg/kg or 0.25 mg/kg). Patients treated with TNK had greater reperfusion rates and better clinical outcomes at 24 h than tPA patients, while no significant differences in intracranial bleeding or other serious adverse events were noted between the groups. EXTEND-IA TNK is a multicenter, randomized trial where patients eligible for thrombectomy were randomized to either IV alteplase (0.9 mg/kg, maximum 90 mg) or tenecteplase (0.25 mg/kg, maximum 25 mg) up to 4.5 hours from onset prior to thrombectomy. The primary outcome measure was reperfusion on the initial catheter angiogram, assessed as modified treatment in cerebral infarction (mTICI) 2 b/3 or the absence of retrievable thrombus. Patients who received TNK achieved higher rates of recanalization than patients who received tPA (22% versus 10%, resp.) with no differences in intracranial hemorrhage (1% in both groups). Although some of these therapies have shown promising results, IV tPA is still recommended as the standard of care. Because of its high fibrin specificity, nonactivation by *β*-amyloid, long half-life, and absence of neurotoxicity, *desmoteplase* is an attractive alternative to tPA for systemic thrombolytic treatment of AIS [47, 48]. Recently DIAS (desmoteplase in acute stroke) assessed the safety and efficacy of desmoteplase given between 3 h and 9 h after symptom onset in patients with occlusion or high-grade stenosis in major cerebral arteries. Treatment with desmoteplase did not improve functional outcomes as measured by modified Rankin Scale of 0–2 at 90 days. Thus, desmoteplase use in the treatment of AIS remains investigational.

*Glycoprotein IIb/IIIa antagonists* prevent platelet aggregation, thereby preventing reocclusion and facilitating thrombus breakdown [49]. In the cardiac literature, in phase IIb studies, these agents have demonstrated improved coronary revascularization in the setting of acute MI, but no significant improvement in the phase III studies [50–52]. Safety of Tirofiban in Acute Ischemic Stroke (SaTIS) was a phase II placebo-controlled study of monotherapy with intravenous tirofiban in patients presenting up to 22 hours after stroke onset. There was no neurological/functional benefit found compared with placebo at 5 months except for lower mortality shown in the treatment group [50, 53]. The subsequent Abciximab in Emergency Treatment of Stroke Trial (AbESTT-II) was a phase III study of GP IIb/IIIa inhibitor monotherapy which was terminated prematurely because of an unfavorable risk-benefit profile in the treatment arm. There was no benefit in neurological recovery in any of the cohorts (within 5-hour onset, between 5 and 6 hours and wake-up strokes) in the abciximab group compared to placebo. Notably, there was a significant increase in symptomatic intracranial hemorrhage [50, 54, 55]. Efficacy and safety of combined intravenous tPA and eptifibatide compared with intravenous tPA alone were investigated in the phase II Combined Approach to Lysis Utilizing Eptifibatide and Recombinant Tissue Plasminogen Activator in Acute Ischemic Stroke-Enhanced Regimen stroke trial (CLEAR-ER) study. The combined treatment group had a lower rate of symptomatic intracranial hemorrhage (2%) and showed a trend towards better functional outcome, with 49.5% achieving mRS 0-1 versus 36% in the standard tPA group [56].

*Argatroban* is a direct thrombin inhibitor which has demonstrated safety in the Argatroban Anticoagulation in Patients with Acute Ischemic Stroke (ARGIS-I) trial [57]. The use of argatroban as an adjuvant to intravenous tPA was investigated in the Argatroban TPA Stroke (ARTTS) study and demonstrated 63% complete recanalization rate at 24 hours [50, 57–63]. In Phase II ARTTS-2 (Randomized Controlled Trial of Argatroban with tPA for Acute Stroke), Barreto et al. conducted a randomized exploratory study to assess safety and the probability of a favorable outcome with adjunctive argatroban and tPA in acute ischemic stroke patients. Patients were treated with standard-dose tPA versus tPA and argatroban (100 *μ*g/kg bolus) followed by infusion of either 1 (low dose) or 3 *μ*g/kg per minute (high dose) for 48 hours. They found that in patients treated with tPA, adjunctive argatroban was not associated with increased risk of symptomatic intracerebral hemorrhage. However, there was no difference in outcomes based on 90-day mRS [64]. Onset to Stroke Treatment Time (MOST) Stroke Trial is a recently funded StrokeNET multicenter multiarm phase 3 clinical trial that will evaluate the benefit of combining either argatroban or eptifibatide with tPA compared to tPA alone in patients with acute stroke.

## 4. Thrombectomy

Initial trials intended to demonstrate the efficacy of endovascular intervention as a potential therapy for acute ischemic stroke were unsuccessful. It was not until recently that its efficacy has been proven.

In 2013, three multicenter prospective randomized controlled trials (RCTs) failed to show a benefit from endovascular intervention for acute ischemic stroke: IMS (Interventional Management of Stroke) III [65], MR RESCUE (Mechanical Retrieval and Recanalization of Stroke Clots Using Embolectomy) [66], and SYNTHESIS Expansion (Intra-arterial versus Systemic Thrombolysis for Acute Ischemic Stroke) [67]. These trials raised concerns about the efficacy of endovascular therapy in large vessel occlusion. However, there were also concerns in the design and conduct of these studies. First, only one of the three trials, MR RESCUE, routinely identified large vessel occlusion with either CTA or MRA. Second, mainly first-generation MT devices were used. Third, patients in the interventional arm of SYNTHESIS Expansion did not receive IV-tPA and were treated in a delayed fashion compared to the medical arm [68]. Considering these limitations, new trials were designed that included the use of second generation stent retriever devices (Solitaire, ev3/Covidien, Trevo, Stryker) that demonstrated significant superior rates of recanalization when compared to the first-generation devices. In 2014, MR CLEAN (Multicenter Randomized Clinical Trial of Endovascular Treatment for Acute Ischemic Stroke in the Netherlands) results were presented which demonstrated significant benefit from endovascular stroke therapy [4]. Following these favorable results, other ongoing trials were stopped early and assessed for efficacy: ESCAPE [5], SWIFT PRIME [6], EXTEND-IA [7], and REVASCAT [8].

MR CLEAN randomized acute stroke patients presenting within 6 hours of stroke onset to standard medical management alone (*n*=267) or standard medical management followed by MT (*n*=233). Eligible patients had a proximal arterial occlusion in the anterior cerebral circulation that (1) was confirmed on vessel imaging and (2) could be treated intraarterially within 6 hours after symptom onset. Retrievable stents were used in 190 of the 233 patients (81.5%) assigned to intra-arterial treatment. There was an absolute difference of 13.5 percentage points (95% CI, 5.9 to 21.2) in the rate of functional independence (modified Rankin score (mRS), 0 to 2) at 90 days in favor of the intervention (32.6% versus 19.1%) [4].

In ESCAPE, 165 patients underwent intervention and 150 were enrolled in the controlled group. 120 in the intervention group and 118 in the control group received IV tPA. In this trial, patients with a proximal intracranial occlusion in the anterior circulation were included up to 12 hours after symptom onset. Patients with a large infarct core or poor collateral circulation on computed tomography (CT) and CT angiography were excluded. In the intervention group, the median time from head CT to first reperfusion was 84 minutes. The rate of functional independence (90-day mRS of 0 to 2) increased with the intervention (53.0%, versus 29.3% in the control group; *P* < 0.001). Intervention was also associated with reduced mortality (10.4%, versus 19.0% in the control group; *P*=0.04) [5].

In SWIFT PRIME, 196 patients (98 patients in each group) underwent randomization into a control group receiving t-PA alone or tPA plus endovascular thrombectomy within 6 hours after symptom onset (intervention group). Patients had confirmed occlusions in the proximal anterior intracranial circulation and an absence of large ischemic-core lesions. Thrombectomy with the stent retriever plus intravenous tPA reduced disability at 90 days over the entire range of scores on the modified Rankin Scale (*P* < 0.001). The rate of functional independence (modified Rankin Scale score, 0 to 2) was greater in the intervention group than in the control group (60% versus 35%, *P* < 0.001) [6].

EXTEND-IA included 70 patients who had received IV tPA within 4.5 hours who were randomized into a control group of receiving IV tPA alone (*n*=35) or to undergo endovascular thrombectomy within 6 hours after the onset of stroke. As in the aforementioned studies, noninvasive vascular imaging was used to identify large vessel occlusion in the anterior circulation. Patients also underwent CT perfusion imaging, which was processed with the use of fully automated software (RAPID) to identify potentially salvageable brain tissue. At 24 hours, the percentage who achieved reperfusion was greater in the mechanical thrombectomy group than that in the IV tPA alone group (median, 100% versus 37%; *P* < 0.001). Also, endovascular therapy improved the functional outcome at 90 days, with more patients achieving functional independence (score of 0 to 2 on the mRS, 71% versus 40%; *P*=0.01) [7].

REVASCAT randomized 206 patients to receive either medical therapy (including IV tPA when eligible) and mechanical thrombectomy (thrombectomy group) or medical therapy alone (control group). All patients had confirmed proximal anterior circulation occlusion that could be treated within 8 hours of symptom onset and had absence of a large infarct on neuroimaging. Initially, exclusion criteria on imaging were evident of a large ischemic core, indicated by an Alberta Stroke Program Early Computed Tomography Score (ASPECTS) of less than 7 on computed tomography (CT) or a score of less than 6 on diffusion-weighted magnetic resonance imaging (MRI). After the enrollment of 160 patients, the inclusion criteria were modified to include patients up to the age of 85 years with an ASPECTS score of more than 8. In this study, thrombectomy reduced the severity of disability over the range of the mRS (adjusted odds ratio for improvement of 1 point, 1.7; 95% confidence interval (CI), 1.05 to 2.8) and led to higher rates of functional independence (mRS 0–2) at 90 days (43.7% versus 28.2%; adjusted odds ratio, 2.1; 95% CI, 1.1 to 4.0) [8].

The PISTE (Pragmatic Ischaemic Thrombectomy Evaluation) was a pragmatic multicenter French clinical trial published in 2017. In this study, 65 patients with anterior circulation LVO who had received IV tPA within 4.5 from stroke onset were randomized 1:1 into groups of patients who received IV tPA alone (control group) and patients who received additional mechanical thrombectomy with a target interval time for IV tPA start to arterial puncture of <90 min. In this study, patients who were candidates for thrombectomy if noninvasive vascular imaging (CTA or MRI) showed occlusion of the intracranial ICA, M1 segment of the MCA, or a single M2 MCA branch. Intervention was to be initiated as quick as possible, and a maximum of 90 min from start of IV tPA to start of the MT procedure was permitted. The primary outcome was the proportion of patients achieving independence defined by a mRS score of 0–2 at day 90. In the intention-to-treat analysis, there was no significant difference in disability-free survival at day 90 with MT (absolute difference 11%, adjusted OR 2.12, 95% CI 0.65 to 6.94; *P*=0.20). Secondary analyses showed significantly greater likelihood of full neurological recovery (mRS 0-1) at day 90 (OR 7.6, 95% CI 1.6 to 37.2; *P*=0.010) [69].

The HERMES collaboration was formed to pool patient data from the first five trials (MR CLEAN, ESCAPE, REVASCAT, SWIFT PRIME, and EXTEND-IA). This meta-analysis concluded that endovascular thrombectomy reduced disability from anterior circulation stroke with LVO, and benefits could be seen in most patients, irrespective of patient characteristics including age or geographical locations [70]. The number needed to treat with endovascular thrombectomy to reduce disability by at least one level on mRS for one patient was 2.6. More importantly, in prespecified subgroup analysis, HERMES revealed that there was a significant benefit in groups that were not eligible for tPA and in a small group of patients who had a large core infarct measured by pretreatment ASPECT scores. These findings represent the foundation of upcoming trials that will evaluate the effect of endovascular therapy in those populations.  Table 2 demonstrates a comparison of these trials.

While these pivotal endovascular trials were in process, an emerging literature suggested that the evolution of ischemic penumbra into the ischemic core and the rate of progression of irreversible injury were highly variable among individuals. This variability is likely mediated by the adequacy of collateral blood flow and the metabolic milieu of stroke patients. Thus, by measuring the individuality of penumbra evolution, the time the window for endovascular therapy could potentially be expanded in selected individuals. DEFUSE 2 demonstrated that outcomes following endovascular thrombectomy differ between patient subgroups based on an MRI profile that suggested that salvageable tissue was present (target mismatch). This study included patients in whom endovascular therapy was anticipated to begin within 12 hours of symptom onset. Patients with target mismatch had greater odds of good functional and radiographic outcomes following reperfusion therapy when compared with patients without target mismatch [71]. In DEFUSE 2, the growth rate of early DWI lesions in these patients was highly variable. A slower rate of DWI growth was associated with a greater penumbral salvage and improved functional outcome following revascularization. These findings suggested that assessing acute infarct growth rates could help identify patients who are most likely to benefit from revascularization [72]. This study created the foundation for the design of two randomized clinical trials of endovascular thrombectomy in patients with a target mismatch profile [71].

The DAWN multicenter randomized trial sought to determine the efficacy of endovascular thrombectomy using the TREVO stent retriever in acute stroke 6–24 hours after symptoms onset. Patients who had evidence of LVO in the anterior circulation on noninvasive vascular imaging (CTA or MRA), who had last been known well 6–24 hours earlier, and who had a determined mismatch between the radiological core infarct measured by an absolute 30% decrease on CBF or DWI and the clinical deficit according to age (<80 years or ≥80 years) were included in the study. Most of the population included patients who did not receive IV tPA because of late presentation. Patients were stratified into three groups: Group A, ≥ 80 years of age, NIHSS ≥ 10, and infarct volume < 21 ml; Group B, < 80 years, NIHSS ≥ 10, and infarct volume < 31 ml; and Group C, < 80 years of age, NIHSS ≥ 20, infarct volume 31 to <51 ml. Infarct volume was processed using RAPID. In each of the three strata, patients were then randomized 1 : 1 into a thrombectomy plus standard medical care (thrombectomy group, *n*=107) or to standard medical care (control group, *n*=107). The trial was stopped early because results of a prespecified interim analysis indicated a high probability of benefit with thrombectomy. The utility-weighted mRS at 90 days was 5.5 in the thrombectomy group versus 3.4 in the control group. The rate of functional independence (mRS 0–2) at 90 days was 49% in the thrombectomy group versus 13% in the control group. Symptomatic intracranial hemorrhage (6% in the thrombectomy group and 3% in the control group, *P*=0.50) and 90-day mortality (19% versus 18%, *P*=1.00) did not differ significantly between the two groups [10]. The number needed to treat to achieve functional independence at 90 days was 2.8.

DEFUSE 3 is the most recent randomized trial assessing thrombectomy in patients beyond 6 hours from last known well. This multicenter study sought to assess the efficacy of mechanical endovascular thrombectomy using second generation stent retrievers and/or aspiration techniques in patients with AIS presenting 6 to 16 hours after they were last known to be well. This trial included patients with proximal anterior circulation LVO, an initial infarct size of less than 70 ml measured by DWI or absolute CBF reduction <30% of normal tissue, and a ratio volume of ischemic tissue on perfusion imaging (defined as *T*_max_ > 6 secs) to infarct volume of ≥1.8. The study was halted early due to efficacy. 182 patients were randomized, 92 patients into the endovascular therapy group and 90 into the medical therapy group. Endovascular therapy plus standard medical therapy was associated with a more favorable distribution of 90-day mRS scores when compared to medical therapy alone (OR, 2.77; *P* < 0.001). Endovascular therapy was also associated with a greater percentage of patients with functional independence (mRS 0–2) at 90 days (45% versus 17%, *P* < 0.001) [11].

When selecting patients for mechanical thrombectomy in patients with AIS onset in <6 hours, current guidelines do not recommend additional neuroimaging beyond CT and CTA or MRI and MRA [9]. This is based on the fact that THRACE and MR CLEAN required only noncontrast CT and demonstration of LVO, and both demonstrated benefit in the treated group [4, 73]. Therefore, criteria based on additional imaging could exclude patients who might benefit from treatment. However, in patients with AIS within 6 to 24 hrs from onset and anterior LVO, additional advanced imaging (CT perfusion, DW-MRI, or MRI perfusion) is recommended to assist in selecting patients for MT based on DAWN and DEFUE 3 criteria [9].

These studies represent a new imaging-based approach for the selection of patients who are most likely to benefit from endovascular thrombectomy. As described by Hacke [74], the usual 6-hour time window for stroke treatment was replaced with a “tissue (viability) window.” These trials represented the bases to the current 2018 AHA guidelines [9].

## 5. Anesthesia for Endovascular Thrombectomy

The best approach to patient sedation, analgesia, and/or anesthesia during endovascular thrombectomy (EVT) has been controversial. This is because most, but not all, observational studies have suggested outcomes that are more favorable when conscious sedation (CS) is used instead of general anesthesia (GA) [73, 75, 76]. The key questions that follow these observations are whether the apparent adverse effect of GA was due to (1) selection bias and/or (2) a process variable (e.g., workflow) or a physiological variable (e.g., blood pressure) related to GA. The answer appears to be “probably yes” to all of these potential explanations.

In terms of selection bias, the great majority of observational studies have reported patients who were selected for GA had greater stroke severity at presentation (e.g., greater NIHSS). Other biases present in many observational studies include (1) a disproportionate assignment of posterior circulation strokes to GA; (2) inclusion of patients who required intubation prior to thrombectomy to GA; (3) inclusion of patients who failed sedation to GA; (4) a greater frequency of proximal (or tandem) occlusions to GA; and (5) a comparison of noncontemporaneous populations (GA patients early in the experience and CS patients later in the experience). Some meta-analyses have attempted to adjust for NIHSS [77], including a recent meta-analysis by Campbell et al. which suggests that GA for EVT was associated with a worse outcome when compared with patients who were not treated under GA. Although these meta-analyses have adjusted for certain baseline variables, other forms of bias remain yet to be explored. Thus, meta-analyses have not entirely provided insight into these questions.

Institutional workflow practices likely contribute to the apparent association between GA and delays in the start of treatment in some observational studies. In the ESCAPE trial, in which only 9% of EVT patients received GA, (1) time between CT scan and arterial puncture was 22 minutes more with GA (RR = 1.43 (95% CI = 1.05–1.93)); and (2) time between arterial puncture and reperfusion was slightly (∼5 minutes), but not significantly, greater with GA (RR = 1.15 (95% CI = 0.77–1.70)) [78]. In contrast, in the SWIFT PRIME trial, in which 36% of EVT patients received GA, neither the time between CT scan and arterial puncture (median 52 minutes) nor the time between arterial puncture and reperfusion (median 32 minutes) was greater with GA; RRs of 0.96 (95% CI = 0.81–1.13), and 0.91 (95% CI = 0.74–1.13), respectively [79]. Thus, it is likely that if, how, and when the anesthesia team is included in the workflow and preparation of the patient prior to EVT is the basis for differences among observational studies regarding treatment delays associated with GA. In particular, when the anesthesia team participates only when a “rescue” is required, GA will appear to be unfavorable both in terms of workflow and outcome. It is also likely that differences among centers in the location of the neurointerventional suite (near versus far from the operating rooms) and availability of the Anesthesia team for emergent procedures can explain some of the apparent delays associated with GA. Nevertheless, if GA is selected, the process of induction of GA and endotracheal intubation unavoidably adds some delay in the onset of treatment. As will be discussed, randomized trials indicate that delay is small, on the order of 10 minutes.

A key determinant of EVT effectiveness is the adequacy of collateral perfusion to the penumbra prior to establishing reperfusion [80, 81]. The most likely reason is that good collaterals result in greater cerebral blood flow (CBF) to the ischemic penumbra [82, 83]. At least in part, collateral flow to the penumbra depends on systemic blood pressure [84]. Because collateral perfusion is so important, it follows that decreases in systemic blood pressure prior to reperfusion may be injurious. This has been observed in two recent observational studies. First, in a subset of 60 GA patients from the MR CLEAN trial, decreases in intraprocedure mean arterial pressure (MAP) were associated with less favorable outcome (mRS) (per 10 mmHg decrease from baseline MAP (which was 100 mmHg) OR = 0.60 (95% CI = 0.43–0.90); *P*=0.03) [85]. In a different study by Whalin et al., all patients underwent EVT with CS (dexmedetomidine) [86]. Patients presented with a MAP = 107 mmHg and functional outcome were associated with all indices of decreased MAP prior to reperfusion. Almost identical to the MR CLEAN results, in patients receiving CS, a decrease in MAP below 100 mmHg decreased the likelihood of good outcome (per 10 mmHg decrease OR = 0.78 (95% CI = 0.62–0.99); *P*=0.043). Thus, with both CS and GA, any substantive decrease in blood prior to reperfusion may be harmful. Outcome differences between CS and GA in some observational studies may be explained, at least in part, because of blood pressure differences between CS and GA [87, 88].

**Figure 3:**
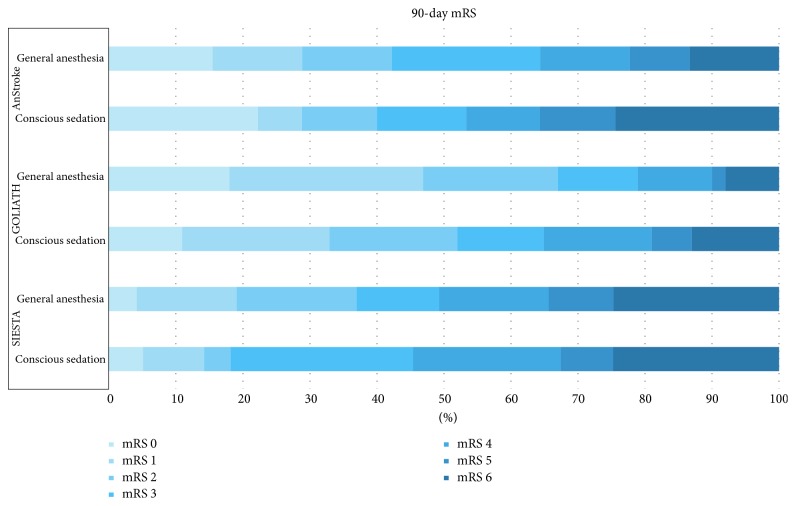
Comparison of randomized clinical trials of general versus conscious sedation for thrombectomy in acute ischemic stroke (mRS at 90 days).

With this background, the findings of three single-center randomized clinical trials (RCTs) of CS versus GA for EVT can be placed in context: SIESTA [89], ANSTROKE [90], and GOLIATH [91]. As summarized in Figure 3, all the three trials found GA to not be associated with less favorable 3-month functional outcomes.

All three trials had similar intraprocedure blood pressure goals: SIESTA (systolic pressure = 140–160 mmHg); ANSTROKE (systolic pressure = 140–180 mmHg); and GOLIATH systolic pressure ≥ 140 mmHg and MAP ≥ 70 mmHg. Most patients, including those receiving CS, required vasopressors to maintain arterial pressure, but with much greater frequency and dosage in patients receiving GA. Nevertheless, in both ANSTROKE and GOLIATH, the minimum value for intra-EVT MAP and the percentage of patients who had >20% decrease in intra-EVT MAP were greater in GA patients. Thus, it is much more difficult to maintain blood pressure at pre-EVT values with GA than with CS.

As summarized in Table 3, in all three RCTs, GA appeared to increase the time between evaluation and arterial puncture by about 10 minutes—an interval consistent with the time required to induce GA and intubate the patient. Good reperfusion was slightly, but not significantly, greater with GA. In SIESTA and ANSTROKE, 14% and 16% of the sedation patients required conversion to GA during EVT, respectively, primarily because of troublesome patient movement. In contrast, in GOLIATH, only 6% of the sedation patients required conversion to GA. Why CS was more successful in GOLIATH than in the other two trials is not obvious. In ANSTROKE, there was a higher incidence of pneumonia in the GA group, while in the CS group, angiographic quality was worse. In SIESTA, the GA group also demonstrated a higher incidence of pneumonia (13.7% versus 3.9%, *P*=0.03), along with hypothermia (32.9% versus 9.1%, *P* < 0.001) and delayed extubation (49.3% versus 6.5%, *P* < 0.001). Despite these findings, none of these studies support the sole use of one technique over the other.

Because these are single-center RCTs, it is not known whether the findings are generalizable. Nevertheless, SIESTA, ANSTROKE, and GOLIATH demonstrate that when (1) GA is integrated into the standard workflow of EVT patients and (2) blood pressure is actively and intensively managed (especially in GA patients), GA does not result in less favorable outcomes than CS. Accordingly, the best evidence indicates that neurointerventional teams can decide to use GA when conditions require it, with less concern that the patient will necessarily be adversely affected. The keys to success with both CS and GA continue to be timely initiation of therapy and support of the penumbra (i.e., blood pressure support) prior to reperfusion. At this time, there is no human data that any specific anesthetic agent or technique is superior to another [92]. An individualized approach, based on patient condition, comorbidities, and expected intraprocedure challenges, appears to be reasonable.

## 6. Postreperfusion Therapy Management

Although guidelines for management of the stroke patient following IV tPA have been established for several years, many of the postprocedural approaches following endovascular thrombectomy remain controversial due to the lack of evidence.

Regardless of the type of reperfusion therapy used, stroke patients should receive intensive neurologic, hemodynamic, respiratory, and metabolic monitoring in a designated stroke or intensive care unit. Stroke patients who received organized care in a stroke unit were more likely to survive, regain independence, and return home when compared to patients who received care in a less organized service or general wards [93].

Hemodynamic support to sustain ischemic penumbral tissue in patients with unsuccessful or partially successful recanalization after reperfusion therapy is essential. However, it is also important to limit the risk of postreperfusion injury and risk of intracerebral hemorrhage (ICH) [94]. Current guidelines recommend that for patients receiving IV tPA and/or mechanical thrombectomy and who have achieved successful reperfusion, it is reasonable to maintain the blood pressure ≤180/105 mmHg [9]. Recanalization rates with IV tPA differ with those with endovascular thrombectomy. In large vessel occlusion stroke, IV tPA results in a recanalization rate that varies between 13% and 45% [16]. On the other hand, mechanical thrombectomy in recent trials has shown successful revascularization (thrombolysis in cerebral infarction score ≥2b) in more than 70% of cases [70]. With this in mind, efforts to increase perfusion with permissive hypertension up to 24–48 hours are commonly practiced in patients who receive IV tPA only [95]. This enables adaptation of the collaterals to accommodate increase blood flow in a durable fashion. In contrast, persistent elevated blood pressures in the setting of near or total recanalization and existing ischemic injury may be harmful [94]. A recent retrospectively analysis of patients who underwent endovascular thrombectomy reported that greater values of systolic blood pressure (SBP) in the first 24 postprocedural are independently associated with greater severity of hemorrhages within 48 hours and worse functional outcomes. Notably, hemorrhage was observed at lower mean values of peak SBP in patients who had successful revascularization compared to those who did not [95]. In hemorrhagic transformation, persistent elevated blood pressure may lead to continued hemorrhage, rebleed, and edema. Therefore, maintaining a SBP <140 or 160 mmHg is reasonable when there is near or total recanalization and/or if there is evidence or suspicion for hemorrhage [94].

The most dreaded complication of thrombolysis is ICH. It typically presents with nausea, vomiting, headache, worsening neurologic deficit, and, in severe cases, with altered level of alertness. In the original NINDS tPA trial, the rate of symptomatic ICH (sICH), defined as the presence of hemorrhage on CT of the head and a decline in neurologic status, was present in 6.4% of those receiving r-tPA and 0.6% in those receiving placebo [31]. Of those patients who suffered sICH in the r-tPA group, approximately 50% died at 3 months. 4.4% of patients had asymptomatic ICH. Major systemic hemorrhages were rare, while minor extracranial hemorrhage occurred in 23% of patients treated with IV-tPA (only 3% in placebo). Risk factors for developing sICH after systemic thrombolysis were hypoattenuation on head CT, elevated serum glucose and history of diabetes, hypertension, increased stroke severity, and protocol violations with treatment outside of the time window [96–99].

Management of sICH after IV tPA usually starts with discontinuation of the tPA infusion followed by immediate noncontrast head CT. A full coagulation panel including fibrinogen and complete blood count are usually ordered. Unfortunately, most patients usually have completed their IV tPA infusion by the time a hemorrhage is detected on CT. There is no proven reversal agent for IV tPA. However, the suggested reversal options include cryoprecipitate (includes factor VIII), tranexamic acid, or aminocaproic acid on a case by case basis.

Another uncommon complication of IV-tPA is angioedema, which occurs in 1–3% of patients. It typically occurs 30–120 minutes after IV tPA infusion. It is thought to be mediated by a similar pathway implicated in angiotensin-converting enzymes (ACEs) and tends to occur contralateral to the infarct. These patients are usually at a high risk of developing the same complication with ACE inhibitors [100]. Treatment involves the administration of diphenhydramine and H2-blockers, followed by IV methylprednisolone or nebulized or subcutaneous epinephrine. In cases of recognition of angioedema IV, tPA should be discontinued, and patients may require endotracheal intubation or even emergent tracheostomy.

Recently, Guidelines from the Society of Neurointerventional Surgery were published to provide guidance in the postprocedural management of a patient undergoing endovascular thrombectomy [94]. According to these guidelines, ICP monitoring has no defined role in LVO since malignant cerebral edema can cause severe clinical deterioration through herniation syndromes despite normal ICP values. Therefore, continuous ICP monitoring does not substitute for clinical and imaging follow-up [101]. Interventions for malignant cerebral edema demonstrated by imaging can include ICP monitoring, head of bed positioning, hyperosmolar agents, hyperventilation, and decompressive craniectomy. Hyperosmolar agents may benefit patients who present cerebral edema following a large volume stroke. Hyperventilation has a short-lived effect (∼1–3 h), and it should be used as a bridging therapy prior to surgical management. Prophylactic hyperventilation however is not recommended. Decompressive craniectomy should be considered in patients who are <60 years of age with large volume strokes who decompensate or who are at imminent risk of decompensating [102]. In patients > 60 years of age, with large volume strokes who decompensate or who are at imminent risk of decompensating, decompressive craniectomy may be considered. However, the mortality benefit may not be followed by functional recovery [103]. EVD placement and suboccipital craniectomy in patients with cerebellar stroke who deteriorate or at imminent risk of decompensating despite medical management may be considered [94].

Finally, given the association with better neurological outcomes, effort should be made to place stroke patients in aggressive rehabilitation facilities [104], and a 90-day follow-up is a reasonable and appropriate standard follow-up in this population [94].

## 7. Conclusion

Substantive advances have been made in the acute management of acute ischemic stroke. Recent trials demonstrating the benefit of endovascular therapy have brought a new era in the treatment of stroke. Now that endovascular thrombectomy has been established as part of the standard of care, further research is needed to continue to optimize existing strategies at prehospital and posthospital care and develop newer methods that incorporate adjunctive emerging reperfusion therapies.

## Figures and Tables

**Table 1:** Inclusion and exclusion criteria for the treatment of acute ischemic stroke with IV tPA within 3 hours from symptom onset.

*Inclusion criteria*
(i) Diagnosis of ischemic stroke causing measurable neurological deficit
(ii) Onset of symptoms <3 h before treatment begins
(iii) Age ≥ 18 y

*Exclusion criteria*
(i) Significant head trauma or prior stroke in the previous 3 months
(ii) Symptoms suggest SAH
(iii) Arterial puncture at noncompressible site in previous 7 d
(iv) History of previous intracranial hemorrhage
(v) Intracranial neoplasm, AVM, or aneurysm
(vi) Recent intracranial or intraspinal surgery
(vii) Elevated blood pressure (systolic > 185 mmHg or diastolic > 110 mmHg)
(viii) Active internal bleeding
(ix) Acute bleeding diathesis, including but not limited to
(x) Platelet count < 100000/mm^3^
(xi) Heparin received within 48 h resulting in abnormally elevated aPTT above the upper limit of normal
(xii) Current use of anticoagulant with INR > 1.7 or PT > 15 s
(xiii) Current use of direct thrombin inhibitors or direct factor Xa inhibitors with elevated sensitive laboratory tests (e.g., aPTT, INR, platelet count, ECT, TT, or appropriate factor Xa activity assays)
(xiv) Blood glucose concentration <50 mg/dL (2.7 mmol/L)
(xv) CT demonstrates multilobar infarction (hypodensity > 1/3 cerebral hemisphere)

*Relative exclusion criteria*
(i) Recent experience suggests that under some circumstances, with careful consideration and weighting of risk to benefit, patients may receive fibrinolytic therapy despite ≥1 relative contraindications. Consider risk to benefit of intravenous tPA administration carefully if any of these relative contraindications is present
(ii) Only minor or rapidly improving stroke symptoms (clearing spontaneously)
(iii) Pregnancy
(iv) Seizure at onset with postictal residual neurological impairments
(v) Major surgery or serious trauma within previous 14 d
(vi) Recent gastrointestinal or urinary tract hemorrhage (within previous 21 d)
(v) Recent acute myocardial infarction (within previous 3 months)

*Note*. Adapted from the AHA study [105].

**Table 2:** Comparison of randomized clinical trials of endovascular thrombectomy in acute ischemic stroke.

RCT	Time window for intervention	Number of patients	Median NIHSS	Median ASPECTS	IV tPA (%)	TICI score 2b/3 (%)	mRS 0–2 at 90 days (%)	sICH (%)	Death rate (%)
MR CLEAN	<6 h from onset	I: 233, C: 267	I: 17, C: 18	I: 9, C: 9	I: 87.1, C: 90.6	59	I: 33, C: 19	I: 7.7, C: 6.4	I: 21, I: 22
ESCAPE	<12 h from onset	I: 165, C: 150	I: 16, C: 17	I: 9, C: 9	I: 72.7, C: 78.7	71	I: 53, C: 29	I: 3.6, C: 2.7	I: 10, C: 19
SWIFT PRIME	<6 h from onset	I: 98, C: 98	I: 17, C: 17	I: 9, C: 9	I: 100, C: 100	88	I: 60, C: 36	I: 0, C: 3.1	I: 9, C: 12
EXTEND-IA	<6 h from onset	I: 35, C: 35	I: 17, C: 13	I: NR, C: NR	I: 100, C: 100	86	I: 71, C: 40	I: 0, C: 5.7	I: 9, C: 20
REVASCAT	<8 h from onset	I: 103, C: 103	I: 17, C: 17	I: 7, C: 8	I: 68, C: 77.7	66	I: 44, C: 28	I: 1.9, C: 1.9	I: 18, C: 16
PISTE	<6 h from onset	I: 33, C: 32	I: 18, C: 14	I: 9, C: 9	I: 100, C: 100	87	I: 57, C: 35	I: 0, C: 0	I: 21, C: 13
DAWN	6–24 h from onset	I: 107, C: 99	I: 17, C: 17	I: NR, C: NR	I: 4.7, C: 13.1	84	I: 49, C: 13	I: 6, C: 3	I: 19, C: 18
DEFUSE 3	6–16 h from onset	I: 92, C: 90	I: 16, C: 16	I: 8, C: 8	I: 11, C: 9	76	I: 45, C: 17	I: 7, C: 4	I: 14, C: 26

RCT: randomized clinical trial; I: intervention group; C: control group; MR CLEAN: Multicenter Randomized Clinical Trial of Endovascular Treatment for Acute Ischemic Stroke in the Netherlands; ESCAPE: Endovascular Treatment for Small Core and Anterior Circulation Proximal Occlusion with Emphasis on Minimizing CT to Recanalization Times; SWIFT PRIME: Solitaire with the Intention for Thrombectomy as Primary Endovascular Treatment; EXTEND-IA: Extending the Time for Thrombolysis in Emergency Neurological Deficits—Intra-Arterial; REVASCAT: Randomized Trial of Revascularization with Solitaire FR Device versus Best Medical Therapy in the Treatment of Acute Stroke due to Anterior Circulation LVO Presenting within Eight Hours of Symptom Onset; PISTE: Pragmatic Ischaemic Stroke Thrombectomy Evaluation; DAWN: DWI or CTP Assessment with Clinical Mismatch in the Triage of Wake-Up and Late Presenting Strokes Undergoing Neurointervention with Trevo; DEFUSE 3: Endovascular Therapy Following Imaging Evaluation for Ischemic Stroke; NIHSS: National Institutes of Health Stroke Scale; ASPECTS: Alberta Stroke Program Early Computed Tomography Score; IV tPA: intravenous recombinant tissue plasminogen activator; TICI: thrombolysis in cerebral infarction; d: day; mRS: modified Rankin Scale; sICH: symptomatic intracranial hemorrhage; NR: not reported.

**Table 3:** Workflow and reperfusion in randomized trials of conscious sedation (CS) versus general anesthesia (GA) for endovascular thrombectomy.

Variable	Trial	CS	GA	*P* value
Time between door,^a^ CT,^b^ and MRI^c^ to arterial puncture (min)	SIESTA^a^	66 ± 20	76 ± 29	0.03
	ANSTROKE^b^	91 (55–123)	92 (68–121)	0.94
	GOLIATH^c^	54 (40–75)	61 (48–73)	0.13

Time between arrival in interventional suite to arterial puncture (min)	ANSTROKE	25 (15–36)	34 (18–47)	0.06
	GOLIATH	15 (12–20)	24 (20–27)	<0.001

TICI 2b/3 reperfusion	SIESTA	62/77 = 81%	65/73 = 89%	0.67
	ANSTROKE	40/45 = 89%	41/45 = 91%	1.00
	GOLIATH	38/63 = 60%	50/65 = 77%	0.04

Values are reported as either mean ± SD, median (interquartile range), or percentage.
